# MW151 Inhibited IL-1β Levels after Traumatic Brain Injury with No Effect on Microglia Physiological Responses

**DOI:** 10.1371/journal.pone.0149451

**Published:** 2016-02-12

**Authors:** Adam D. Bachstetter, Zhengqiu Zhou, Rachel K. Rowe, Bin Xing, Danielle S. Goulding, Alyssa N. Conley, Pradoldej Sompol, Shelby Meier, Jose F. Abisambra, Jonathan Lifshitz, D. Martin Watterson, Linda J. Van Eldik

**Affiliations:** 1 Sanders-Brown Center on Aging, University of Kentucky, Lexington, Kentucky, United States of America; 2 Spinal Cord and Brain Injury Research Center (SCoBIRC), University of Kentucky, Lexington, Kentucky, United States of America; 3 Department of Anatomy and Neurobiology, University of Kentucky, Lexington, Kentucky, United States of America; 4 Department of Physiology, University of Kentucky, University of Kentucky, Lexington, Kentucky, United States of America; 5 Department of Pharmacology, Northwestern University, Chicago, Illinois, United States of America; University of South Florida, UNITED STATES

## Abstract

A prevailing neuroinflammation hypothesis is that increased production of proinflammatory cytokines contributes to progressive neuropathology, secondary to the primary damage caused by a traumatic brain injury (TBI). In support of the hypothesis, post-injury interventions that inhibit the proinflammatory cytokine surge can attenuate the progressive pathology. However, other post-injury neuroinflammatory responses are key to endogenous recovery responses. Therefore, it is critical that pharmacological attenuation of detrimental or dysregulated neuroinflammatory processes avoid pan-suppression of inflammation. MW151 is a CNS-penetrant, small molecule experimental therapeutic that restores injury- or disease-induced overproduction of proinflammatory cytokines towards homeostasis without immunosuppression. Post-injury administration of MW151 in a closed head injury model of mild TBI suppressed acute cytokine up-regulation and downstream cognitive impairment. Here, we report results from a diffuse brain injury model in mice using midline fluid percussion. Low dose (0.5–5.0 mg/kg) administration of MW151 suppresses interleukin-1 beta (IL-1β) levels in the cortex while sparing reactive microglia and astrocyte responses. To probe molecular mechanisms, we used live cell imaging of the BV-2 microglia cell line to demonstrate that MW151 does not affect proliferation, migration, or phagocytosis of the cells. Our results provide insight into the roles of glial responses to brain injury and indicate the feasibility of using appropriate dosing for selective therapeutic modulation of injurious IL-1β increases while sparing other glial responses to injury.

## Introduction

Traumatic brain injury (TBI), a common cause of morbidity and mortality, initiates a cascade of pathophysiological events that can exacerbate the primary injury and worsen long-term outcome, including an increased potential for neurodegenerative complications. The overproduction of proinflammatory cytokines, presumably produced by glia, is one of the secondary events that contributes to worsening neurological outcomes in many central nervous system (CNS) disorders, including TBI (for recent reviews see [1, 2]). Further, cytokine responses after injury show a delayed temporal window, with a peak cytokine response in rodents and humans occurring many hours after the injury [3–8]. Moreover, various aspects of neuroinflammation can persist for months to years in animal models and humans [3, 9–11]. The extended time window, and the contribution to pathophysiology progression, renders attenuation of proinflammatory cytokine overproduction a viable aspect of the neuroinflammation process amenable to therapeutic intervention.

To address the need for small molecule CNS therapeutics for TBI and neurodegenerative disease, we developed CNS-penetrant, small molecule experimental therapeutics targeting neuroinflammation [12–15]. One of these compounds, MW01-2-151WH (= MW151) is a unique chemical entity that is a potential first-in-class candidate for addressing the challenge of selective modulation of glia responses [13]. MW151 was developed using the classic functional approach that is unbiased and has had historical success in delivering widely used drugs to clinical practice. The focus was on a deliverable with attractive pharmacological properties and activity as a selective suppressor of stressor-induced up-regulation of neuroinflammatory responses of activated glia such as proinflammatory cytokine overproduction [13]. Our discovery process began with a validated drug discovery engine and a mouse model we established with defined time windows for proinflammatory cytokine production, onset of synaptic dysfunction and cognitive behavior deficits [16–18]. The novel small molecules were designed using pharmacoinformatics and a curated database of stable CNS-penetrant drugs in conjunction with a scaffold hopping medicinal chemistry approach. Viable hits were subjected to focused refinement to improve absorption-distribution-metabolism-excretion-toxicity (ADMET)-related features. One of the best of class compounds emerging from the hierarchal pharmacological filtering process was MW151, an experimental therapeutic with excellent chemical and pharmacokinetic properties. MW151 is a water-soluble, chemically stable, small molecule that is orally bioavailable and CNS-penetrant, with a brain:blood ratio >1, similar to or better than CNS drugs in clinical use or under development. MW151 is metabolically stable (>90% remaining after 2hr) in human hepatocytes; its unusual stability in this standard assay is attractive for several reasons, including less probability of metabolite toxicity or adverse pharmacology. MW151 also has high potential for safety, with no detectable histological liver toxicity at chronic low doses or acute high doses and no evidence of cardiovascular toxicity as assessed by prolongation of QTc interval.

MW151 is selective in its action and is not a pan-suppressor of neuroinflammation. For example, MW151 suppresses disease- and injury-induced overproduction of proinflammatory cytokines such as interleukin-1 beta (IL-1β) and tumor necrosis factor alpha (TNFα), but does not block anti-inflammatory cytokines such as interleukin-10 (IL-10) [19]. The pharmacological mechanism of action of MW151 is one that restores activated pathways back towards homeostasis, as shown by its lack of effect in control animals and its failure to depress basal cytokine levels at efficacious doses. MW151’s selective alteration of up-regulated biosynthetic processes such as proinflammatory cytokine production allows the potential of an extended pharmacodynamic effect compared to the time course of detectable drug levels in the target tissue. The animal model data are consistent with this potential. For example, MW151 has been shown to selectively restore injury- or disease-induced overproduction of proinflammatory cytokines towards homeostasis and improve neurologic outcomes in a variety of animal models of diseases where proinflammatory cytokine overproduction is a component of pathophysiology progression [3, 4, 19–26].

In closed head injury (CHI) model of TBI, acute administration of low dose (1 to 10 mg/kg) MW151 during a limited time coincident with increasing cytokine production (hrs post-injury) leads to improvements in neurologic endpoints evidenced weeks later [4, 20]. Because brain injury is a heterogeneous disorder [27] with direct and indirect injuries that vary with the type and extent of injury, it was important to test the effects of MW151 in a different type of brain injury. The current study was specifically designed to determine if MW151 would be effective at suppressing a prototypical proinflammatory cytokine (IL-1β) response in the mFPI model of TBI, a diffuse axonal injury model that is of greater injury severity than the CHI model. We then examined cellular mechanisms in activated glia to determine if MW151 was able to bring about the therapeutically relevant suppression of the IL-1β cytokine response at concentrations that leave intact glia proliferation, migration, and phagocytosis. The results demonstrate the selective glia effects of MW151 and add to the accumulating body of evidence that supports new therapeutic approaches to CNS disease that target glia pathophysiology mechanisms with retention of restorative functions through the use of appropriate drug selection and dosing.

## Materials and Methods

### Animals

The Institutional Animal Care and Use Committee (IACUC) of the University of Kentucky approved the use of animals in this study (IACUC Protocol Number: 2011–0869), which were conducted in accordance with the principles of animal care and experimentation in the Guide For the Care and Use of Laboratory Animals. All experiments used adult (20-30g) male C57BL/6 mice obtained from Harlan Laboratories (Indianapolis, IN). The animals were housed in a 14 h light/10 h dark cycle at a constant temperature (23°C±2°C) with food and water available ad libitum. Following NIH guidelines [28, 29], experiments included randomization of animals, and blinding of treatment groups and tissue samples.

### Midline Fluid Percussion Injury (mFPI)

The mFPI was conducted as previously described [5, 6, 30]. Briefly, mice were anesthetized with isoflurane, which was continuously delivered via nosecone during surgery. Body temperature was maintained using a Deltaphase isothermal heating pad (Braintree Scientific Inc., Braintree, MA). A midline craniotomy was performed via trephination (3 mm outer diameter) midway between bregma and lambda. An injury hub consisting of a modified Luer-Lock hub (BD Biosciences) was affixed using cyanoacrylate gel and dental acrylic (Hygenic Corp., Akron, OH). Mice were given 16–24 h to recover from the craniectomy before the mice were re-anesthetized with isoflurane, and the injury hub was attached to the male end of the fluid percussion device (Custom Design and Fabrication, Virginia Commonwealth University, Richmond, VA). An injury of moderate severity (1.2 ± 0.05 atm) was administered by releasing the pendulum onto the fluid filled piston. Sham mice were subjected to all the experimental and a surgical condition described above, except that the pendulum was not released onto the piston, thus, a fluid percussion injury was not administered. The injury hub was removed and the brain was inspected for uniform herniation and integrity of the dura. Mice were excluded from the study if the integrity of the dura was in question. A potential consequence of the traumatic brain injury is death. Approximately 10% of brain-injured animals died as a result of the injury, primarily from respiratory distress or pulmonary edema. Death post-injury occurs from seconds—to a few minutes after the injury. No post-operative analgesics were administered. The IACUC protocol (2011–0869) stipulated the following clinical signs to be used to determine when early/humane euthanasia of the animal was required; specifically, if any mouse displayed abnormal behaviors or appearance, including: rapid weight loss (15–20 percent within a few days), loss of ability to ambulate (inability to access food or water), labored respiration, or infection. If these clinical symptoms were observed, the veterinary services were to be contacted for recommendations on treatment options or need for euthanasia. No adverse events were seen which required early/humane euthanasia.

### Synthesis and use of MW151

**Fig 1:**
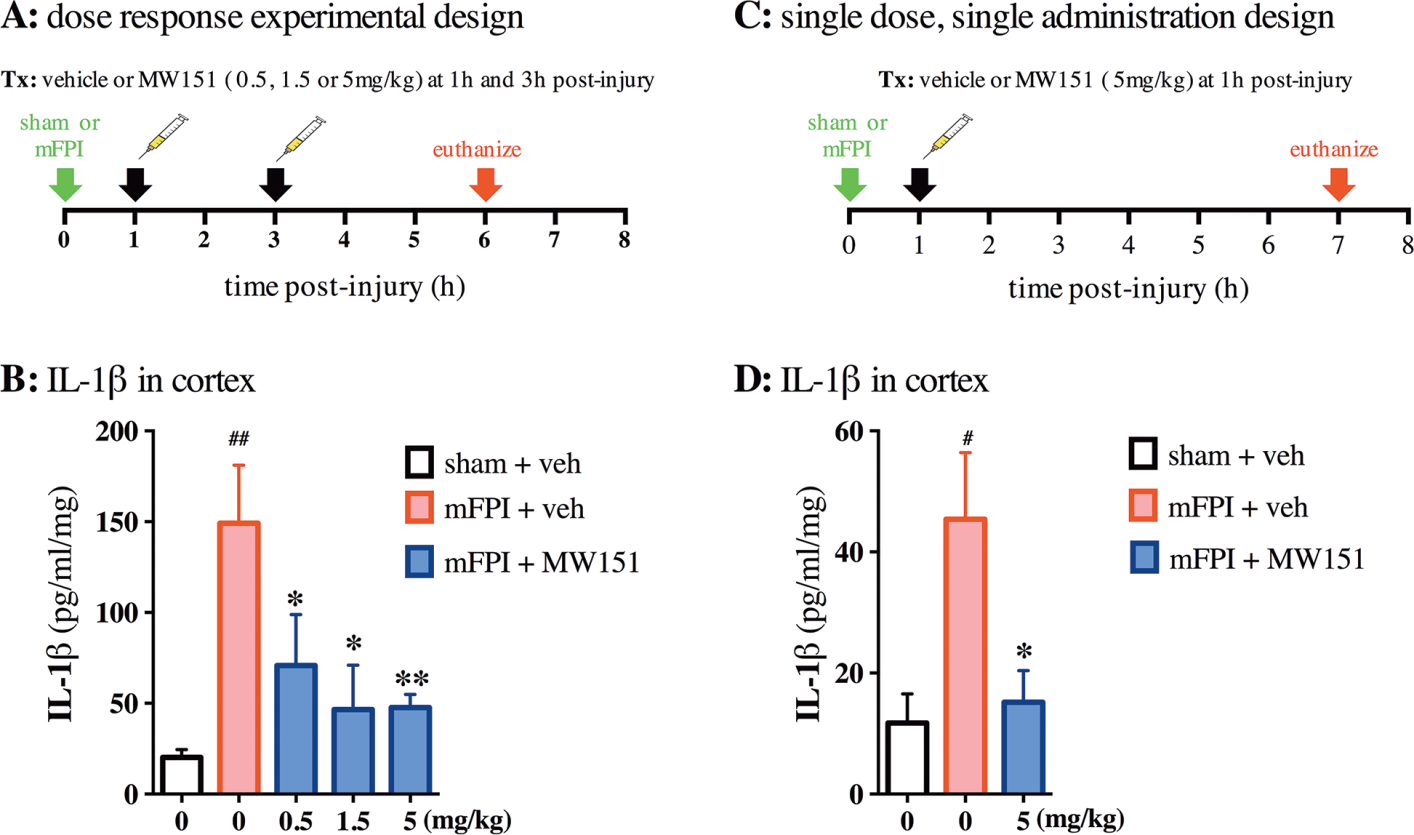
Effects of MW151 on suppression of diffuse brain injury-induced IL-1β in the cortex. **(A)** Overview of experimental design for dual administration, dose response experiment. **(B)** IL-1β was increased in the mFPI + veh group compared to sham + veh, and MW151 suppressed the injury-induced IL-1β increase at the three doses tested (F_4,39_ = 5.4895; p = 0.0013) (n = 8 sham + veh; n = 12 mFPI + veh; n = 6 mFPI + MW151 0.5mg/kg; n = 6 mFPI + MW151 1.5mg/kg; n = 12 mFPI + MW151 5mg/kg). **(C)** Overview of experimental design for single administration, single dose experiment. (**D**) IL-1β was increased in the mFPI + veh group compared to sham + veh, and MW151 suppressed the injury-induced IL-1β increase compared to mFPI + veh (F_2,14_ = 3.8882; p = 0.0499) (n = 3 sham + veh; n = 7 mFPI + veh; n = 5 mFPI + MW151 5mg/kg). ^#^p<0.001 compared to sham + veh. *p<0.05, **p<0.001 compared to mFPI + veh. (mFPI = midline fluid percussion injury; veh = vehicle).

MW01-2-151SRM (2-(4-(4-methyl-6-phenylpyridazin-3-yl)piperazin-1-yl)pyrimidine) was synthesized and characterized as previously reported [13]. For *in vivo* and *in vitro* experiments, MW151 was dissolved in 0.9% sterile NaCl (saline: Hospira NDC 0409-4888-10). MW151 (0.5–5.0 mg/kg) or saline control was administered *in vivo* by intraperitoneal injection as previously described [3, 4, 19]. See Fig 1 for an outline of dosing.

### Brain tissue harvesting, biochemical and histological endpoints

Mice were euthanized by sodium pentobarbital overdose and transcardially perfused with ice-cold phosphate buffered saline (PBS) for 5 min, then the brains were rapidly removed and dissected, as previously described [3, 4, 19]. IL-1β levels were measured in brain homogenates using Meso Scale Discovery (MSD) ELISA, as previously described [3, 4, 19]. Immunohistochemistry (IHC) staining was done on every 12^th^ section starting from 1mm to -3mm from Bregma following established methods, and quantified using the Aperio ScanScope XT digital slidescanner and Aperio ImageScope software positive pixel count algorithm (version 9), as previously described [3, 4, 19]. Primary antibodies used included: rabbit anti-GFAP (Dako Cat#Z0334; (1:10,000)); rabbit anti-IBA1 (Wako Cat#019–19741; (1:10,000)); and rat anti-F4/80 (MCA497GA) (AbD Serotec scientific Cat#MCA49247GA; (1:20,000)); and rabbit anti-pSTAT3 (Cell Signaling Cat #9145, (1:500)). Immunofluorescence staining was done following established methods as previously described [31]. Antibodies used included: rabbit anti-IBA1 (Wako Cat#019–19741; (1:2,000), and Alexa 488 goat anti-rabbit IgG (ThermoFisher Scientific Cat# A-11034; 1:200). Immunofluorescent images were taken on a Nikon C2Plus Confocal Microscope using a 40x objective, a 0.5μm step size, 1x zoom, 2048 x 2048 pixels. Imaris software (version 8.1.2: Bitplane AG, Zurich Switzerland) was used for 3D reconstructions of the confocal Z-stacks.

### BV-2 microglia cell line assays

The murine microglial immortalized BV-2 cell line, originally derived by Dr. Elisabetta Blasi [32], is a well-established cell line that mimics many responses of activated microglia. BV-2 cells (passage 25–35) were cultured in DMEM/F12 (Mediatech; Cat no. #15-090-CV) supplemented with 10% FBS, 100 IU/ml penicillin, 100 μg/ml streptomycin (Mediatech Cat no. 30-002-CI) and 2mM L-Glutamine (Mediatech Cat no. 25-005-CI), as previously described [33]. Incucyte Zoom (Essen Bioscience) live cell imager and Incucyte Zoom software were used for growth curve, migration and phagocytosis assays. The Wound Maker (Essen Bioscience) was used for the migration assay. For the phagocytosis assay, pHrodo red *E*. *coli* Bioparticles (ThermoFisher Scientific Cat# P35361) was added to the wells at a final concentration of 400μg/ml. Cytochalasin D (CytD; Sigma Cat# C8273; 10mM), an inhibitor of actin polymerization, was used as a positive control. CytD was dissolved in dimethylsulfoxide (DMSO). A DMSO control was included in all experiments. As no difference was found between the saline control and the DMSO control, only the saline control is shown. For STAT3 assays, BV-2 cells were treated with interferon gamma (IFNγ) (10μg/ml; R and D systems; Cat#485-MI) or IL-6 (1ng/ml; R and D systems; Cat#406-ML) for 60 min in the absence or presence of increasing concentrations of MW151. Levels of pSTAT3 and total STAT3 were measured in cell lysates by MSD ELISA or western blot following previously described methods [12, 33]. The following primary antibodies from Cell Signaling Technology (Beverly, MA) were used for the western blot assay: rabbit anti-pSTAT3 (cat. no. 9131 (1:1000)); rabbit anti-total STAT3 (cat no. 9232 (1:1000)); mouse anti-β-Actin (Cat no. 3700 (1:10000)). Quantitative western blot analysis was done using the Li-Cor Odyssey Infrared imager.

### Statistics

JMP Software version 10.0 was used for statistical analysis. A one-way ANOVA was used to examine differences across groups. A two-tailed Student’s T test was used for post hoc analysis to compare only the effect of injury compared to sham, and to compare the effect of MW151 compared to vehicle in the injured mice, as these comparisons were determined *a priori* to be the ones of interest. Differences between means were considered significant at α = 0.05. Graphs were generated using GraphPad Prism software version 6.0, and values are expressed as mean ± SEM.

## Results

### MW151 suppresses IL-1β levels in the cortex after midline fluid percussion brain injury in mice

In order to extend the significance of the dose-dependent modulation of brain injury induced increases in IL-1β levels we have previously reported following CHI [3, 4, 20], we examined MW151 treatment in a mFPI model (**Fig 1A)**. The mFPI model is associated with diffuse axonal injury [34, 35] and is also of greater injury severity than the CHI model. An important consideration for dosing with an experimental therapeutic is to treat during the time window when the targeted mechanism of action, IL-1β levels in this case, is undergoing change. In the mFPI model, the increase in IL-1β levels begins by 1h and peaks 6–9h post injury [5, 6]. Therefore, we administered MW151 at 1h and 3h post-injury in order to target IL-1β as its brain cortex levels are increasing after injury. One of three doses (0.5, 1.5 and 5.0 mg/kg) was administered at each time point. Mice were euthanized at 6h post-injury, in order to ensure that the protein levels of IL-1β would be reproducibly elevated in the mFPI + vehicle (veh) -treated mice. This insures sufficient assay sensitivity to evaluate a dose response curve. As expected based on prior studies, IL-1β levels at 6h post-injury were increased (p = 0.0002) in the mFPI + veh mice compared to the sham + veh mice (**Fig 1B**). MW151 treatment during this time window suppressed the injury-induced IL-1β levels. The inhibition was significant at all three doses compared to mFPI + veh mice: 0.5 mg/kg (p = 0.0283), 1.5 mg/kg (p = 0.0049), and 5 mg/kg (p = 0.0008) (**Fig 1B**). Maximal suppression was obtained at 1.5 mg/kg, with no further suppression at 5.0 mg/kg.

To test whether a single administration of MW151 at a maximal dose could suppress IL-1β levels, mice were administered MW151 (5mg/kg) at 1h post-injury and cytokine levels measured 6h hours later, which is 7h post-injury (**Fig 1C).** The levels of IL-1β were elevated in the mFPI + veh -treated mice compared to the sham + veh -treated mice (p = 0.0459; **Fig 1D**). MW151 administration (5mg/kg) at 1h post-injury suppressed IL-1β levels compared to the control -treated group (p = 0.0362). In the control (mFPI + veh -treated) mice, the level of IL-1β in the cortex at 7h post-injury (**Fig 1D**) was only about 1/3 the level in the cortex at 6h post-injury experiment (**Fig 1B**).

### MW151 treatment following diffuse traumatic brain injury does not alter glial morphological responses

**Fig 2:**
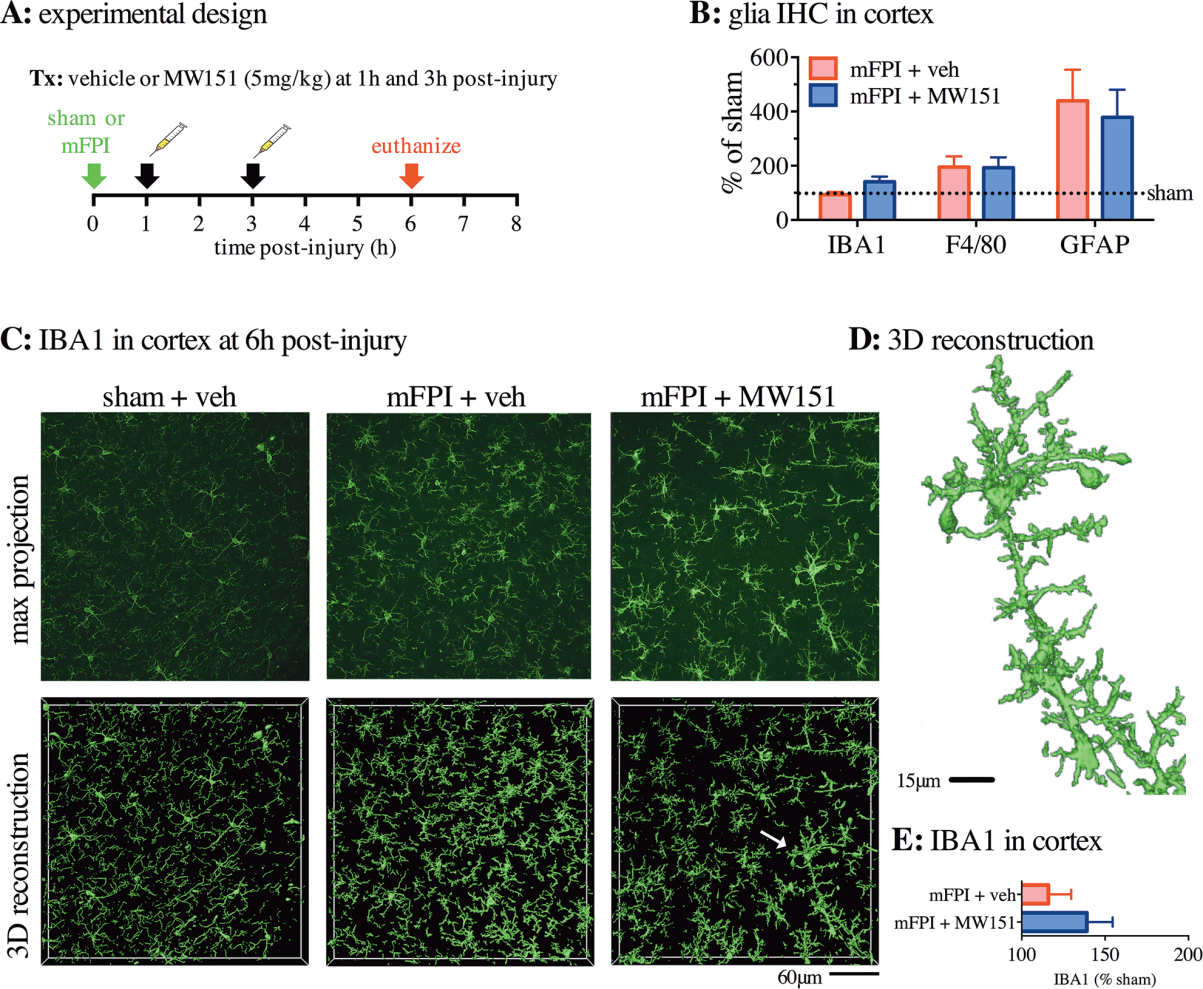
No effects of MW151 on glia morphological changes in the cortex after diffuse brain injury. **(A)** Overview of experimental design. **(B)** Digital neuropathological assessment of IBA1, F4/80, and GFAP IHC in the cortex was done using the Aperio ScanScope and positive pixel algorithm (n = 2 sham + veh, n = 9 mFPI + veh, n = 10 mFPI + MW151). **(C)** Maximal intensity projections (max projection) and 3D reconstructions of Z-stacks taken on confocal microscope showing microglia from the cortex of mice following a diffuse brain injury. White arrow indicates IBA1^+^ profile of 3D reconstruction that is shown in isolation and at higher magnification in **(D)**. **(E)** Quantification of the microglia volume in the 3D reconstruction normalized to the total volume of the Z-stack shows the increase in IBA1^+^ microglia volume in the mFPI mice compared to the sham-treated mice (n = 3 sham + veh, n = 6 mFPI + veh, and n = 6 mFPI + MW151). (mFPI = midline fluid percussion injury; veh = vehicle).

In order to explore the potential for MW151 to attenuate stressor-induced upregulation of IL-1β production while retaining normal glial functions associated with homeostasis, we examined the effect of MW151 on biomarkers of glial *in vivo* status. Specifically, IBA1 and F4/80, which are macrophage / microglia markers (abbreviated microglia from here on), and one astrocyte marker (GFAP) were used. IHC staining was done in brain sections from mice that had been treated at 1h and 3h post-injury with 5mg/kg MW151 and euthanized at 6h post-injury (**Fig 2A**). This is the same treatment paradigm as in Fig 1A where we see maximum injury-induced IL-1β levels and suppression by MW151. Compared to sham + veh -treated mice, the mFPI injury induced a 95% ± 78% increase in F4/80 and a 339% ± 253% increase in GFAP staining, but no difference was found between the mFPI + veh mice compared to the mFPI + MW151 -treated mice (**Fig 2B**). A non-significant increase (47% ± 21%) in IBA1^+^ staining was seen in the mFPI + MW151 -treated mice compared to the mFPI + veh -treated mice (**Fig 2B**). To further explore the potential subtle microglia difference, brain sections were stained with IBA1, imaged using confocal microscopy, and microglia morphology assessed using Imaris 3D reconstruction (**Fig 2C and 2D**). Quantification of the Imaris 3D reconstruction of the IB1+ staining confirmed the slight increase in IBA1^+^ staining in the mFPI + MW151 -treated mice compared to the mFPI + veh -treated mice (**Fig 2E**). The functional significance of this difference during a repeat administration of a MW151 maximal dose is not known. The difference does not correlate with any adverse pharmacological events, but warrants potential investigation in future studies of dosing regimens for acute brain injury.

### MW151 treatment does not impair microglial proliferation, migration, or phagocytosis

**Fig 3:**
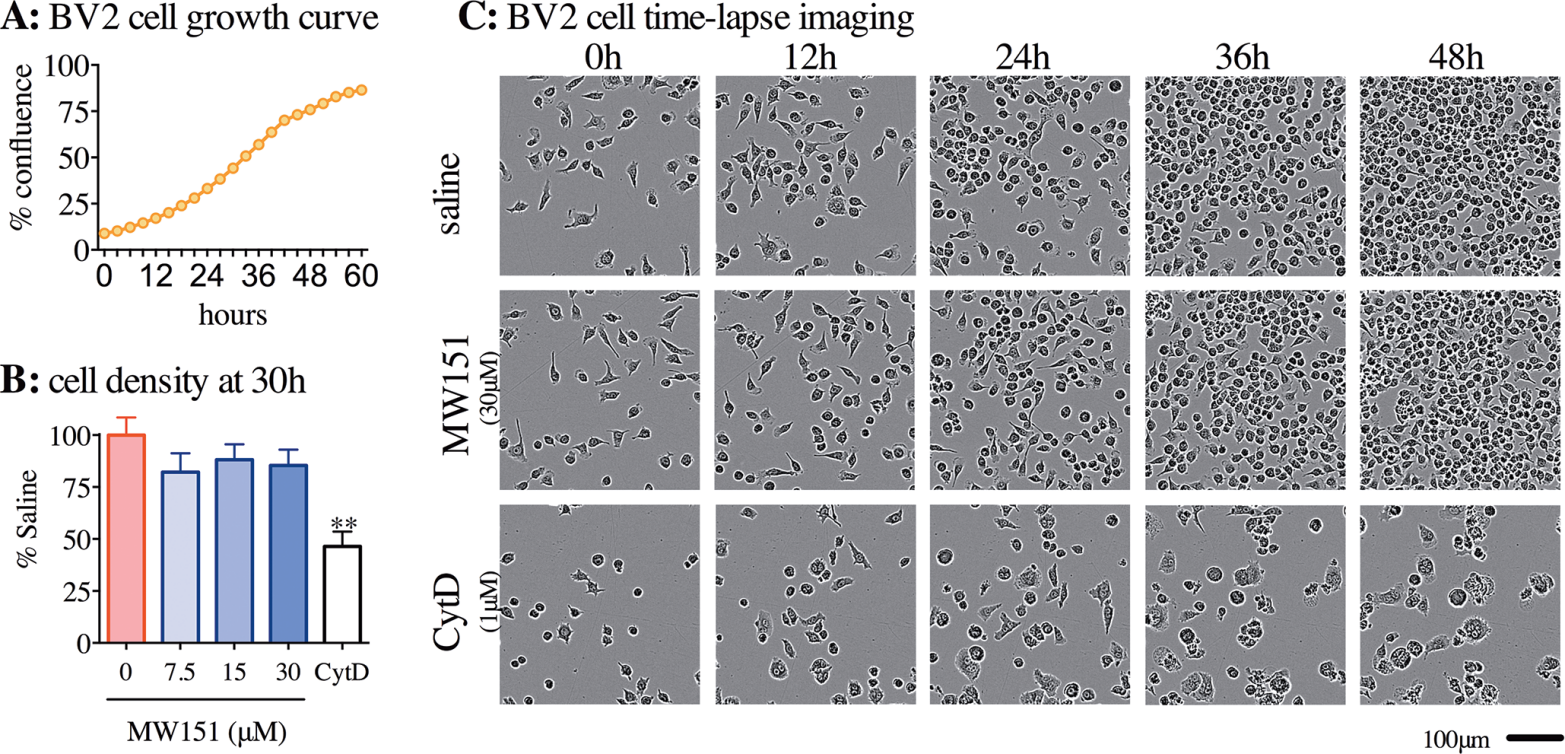
No effects of MW151 on BV-2 microglia cell growth curves. **(A)** Representative example of BV-2 cell growth curve over the first 60h after plating in a 96 well plate at 5,000 cells/well. **(B)** BV-2 cells were treated at 6h after plating, with vehicle control (saline), MW151 (7.5, 15, or 30μM), or cytochalasin D (CytD, 1 μM). Each experiment was carried out in 8 replicates, with graph summarizing 3 independent experiments (mean ± SEM, n = 3). **p<0.01 compared to saline. **C)** Representative photographs of BV-2 cells treated with saline, 30μM MW151, or 1μM CytD at 0, 12, 24, 36 and 48 hrs after drug treatment. All imaging was done using Incucyte Zoom at 10x objective.

In order to probe the selective action of MW151 on microglial activities, we examined three well-established physiological responses of microglia, namely, proliferation, migration and phagocytosis. These were assessed using live cell imaging of BV-2 cells, with or without MW151 treatment. **Fig 3A** illustrates the growth curve of BV-2 cells over 60h, starting with the cells at approximately 10% confluency, and measured using Incucyte Zoom live-cell imager (Essen Bioscience). BV-2 cells were treated with MW151 to determine the effect of compound treatment on the BV-2 cell growth curve. A small decrease in cell density was seen at the 30h timepoint after MW151 treatment, but the decrease was not significant and was not concentration-dependent (**Fig 3B)**. Representative photomicrographs over the first 48h after drug treatment show no change in BV-2 cell morphology in the MW151 group compared to the vehicle (saline) control group (**Fig 3C**). As expected, the positive control, cytochalasin D (CytD), caused a significant decrease in proliferation and induced an abnormal cell morphology (**Fig 3B and 3C**).

**Fig 4:**
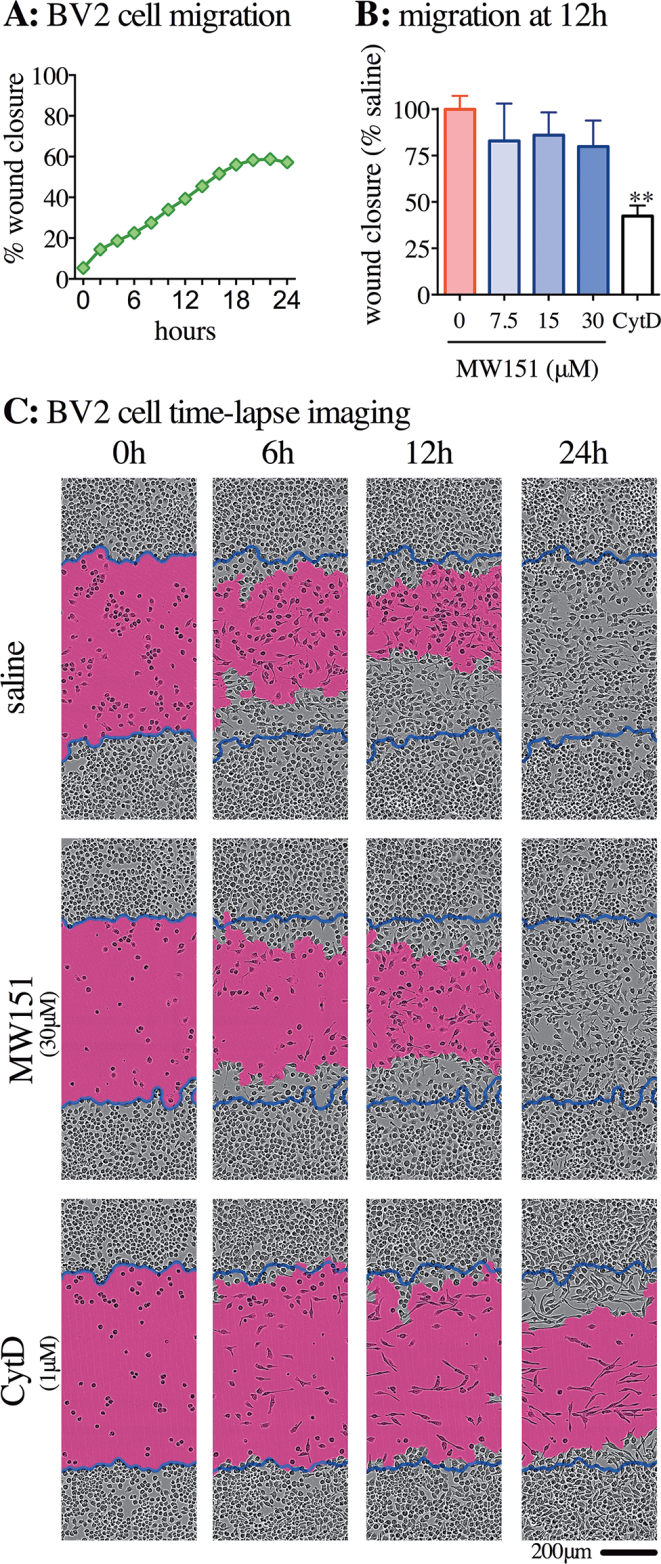
No effects of MW151 on BV-2 microglia cell migration into scratch wound. **(A)** Representative graph of the rate of BV-2 cell migration into wound area, as determined by the percent confluency in the area left nearly devoid of cells after the scratch wound, and plotted as percent wound closure. **(B)** At 12h, during the linear phase of the wound closure, the effect of vehicle control (saline), MW151 (7.5, 15, or 30μM), or cytochalasin D (CytD, 1 μM) was quantified, as in (A), and plotted as percent of saline vehicle. The graph represents the average of three independent experiments (mean ± SEM, n = 3), each experiment carried out in 8 replicates for each treatment. **p<0.01 compared to saline vehicle. **(D)** Representative photographs of BV-2 cells migrating into scratch wound area with saline, 30μM MW151 or 1μM CytD treatment at 0, 6, 12, and 24 hrs after initial scratch. Blue lines indicate initial scratch wound area. Pink is wound area at each time point, calculated by Incucyte Zoom software. Images and data obtained using Incucyte Zoom at 10x objective.

Cell migration activity was screened using a scratch wound assay that measures the ability of treated cells to migrate into an empty space created by a cell layer wound. BV-2 cells were plated in a 96 well plate and a scratch wound was made on all 96 wells simultaneously using the Essen Bioscience WoundMaker when the cells were approximately 90% confluent. The wells were imaged every two hours over the next 24 hours to determine the rate at which the BV-2 cells filled the gap made by the scratch wound. An increase in the number of BV-2 cells migrating into the scratch wound was seen until 18h post-scratch, at which time the increase in the number of cells over time plateaued (**Fig 4A**). Similar to the cell growth assay in **Fig 3**, a detectable but non-significant decrease in the confluency of the cells in the wound area was observed at 12h (**Fig 4B**). The change, however, was not drug concentration-dependent. As shown in **Fig 4C**, no marked difference was seen with MW151 treatment compared to saline control over the 24h time period that was recorded. In contrast, treatment with the CytD positive control led to a noticeable reduction in the number of cells that entered into the wound area.

**Fig 5:**
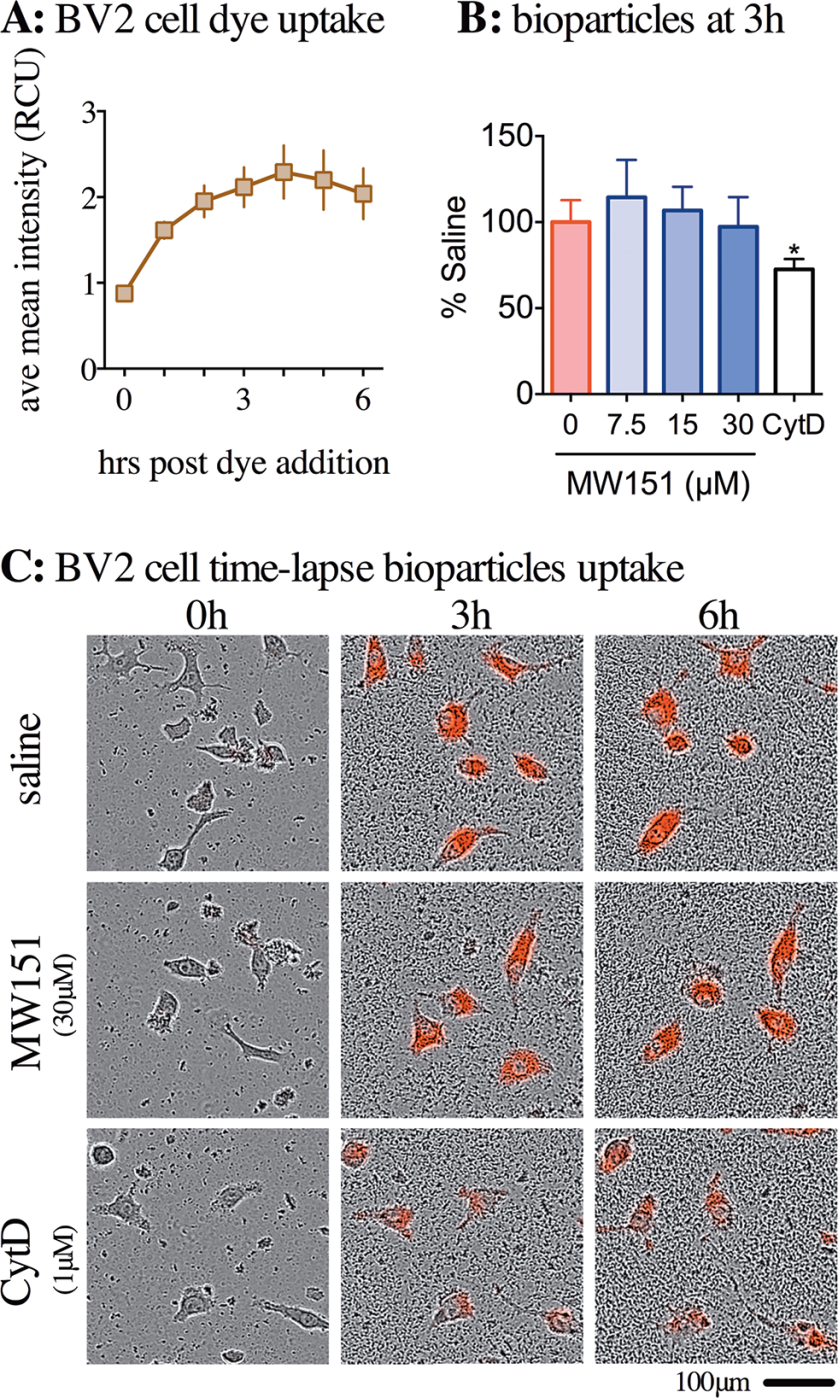
No effects of MW151 on BV-2 microglia cell engulfment of pH sensitive *E*. *coli* bioparticles. **(A)** The pHrodo dye is non-fluorescent at neutral pH, but acidification, presumably in the cell phagosome, causes the dye to fluoresce in the red spectrum. Over the first 3h after adding the pHrodo-labeled bioparticles, the average mean intensity of the red calibrated unit (RCU) increased, but after 3h the RCU intensity plateaued. **(B)** At 3h, near the end of the linear phase of increasing RCU, the effect of treatment with vehicle control (saline), MW151 (7.5, 15, or 30μM), or cytochalasin D (cytD, 1 μM) was quantified. The graph represents the average of three independent experiments (mean ± SEM, n = 3), each experiment carried out in 4 replicates for each treatment. **p<0.05 compared to saline vehicle. **(C)** Representative photographs of BV-2 cells treated with saline, 30μM MW151 or 1μM CytD treatment at 0, 3, and 6 hrs after the addition of the bioparticles. Images and data obtained using Incucyte Zoom at 20x objective.

Phagocytosis was the third microglia physiological response investigated using the BV-2 cell line and live-cell imaging. Cells were plated at 5000 cells/well and pH-sensitive *E*. *coli* bioparticles were added to the wells after 14–16h, when the cells were approximately 10% confluent. The bioparticles are labeled with a pHrodo dye that is non-fluorescent at neutral pH but fluoresces in the red spectrum at acid pH. The assay takes the fluorescence change as an indicator of acidification reflective of cell phagosome internalization. As shown in **Fig 5A**, BV-2 cells internalized the bioparticles during the first 3h after addition to the wells. To determine if MW151 had an effect on bioparticle uptake, three concentrations of MW151 (7.5, 15, or 30μM) were added 30 min prior to the addition of bioparticles to the well. These read outs for the saline- and MW151-treated cells were indistinguishable (**Fig 5B and 5C)**. Although an incremental increase at the lower MW151 concentrations could be observed in this experiment, it was not statistically significant. The lack of significant change with MW151 treatment stands in contrast to the CytD positive control (p = 0.0284) (**Fig 5B and 5C**).

### Probing potential molecular reporters of MW151 action in activated glia

**Fig 6:**
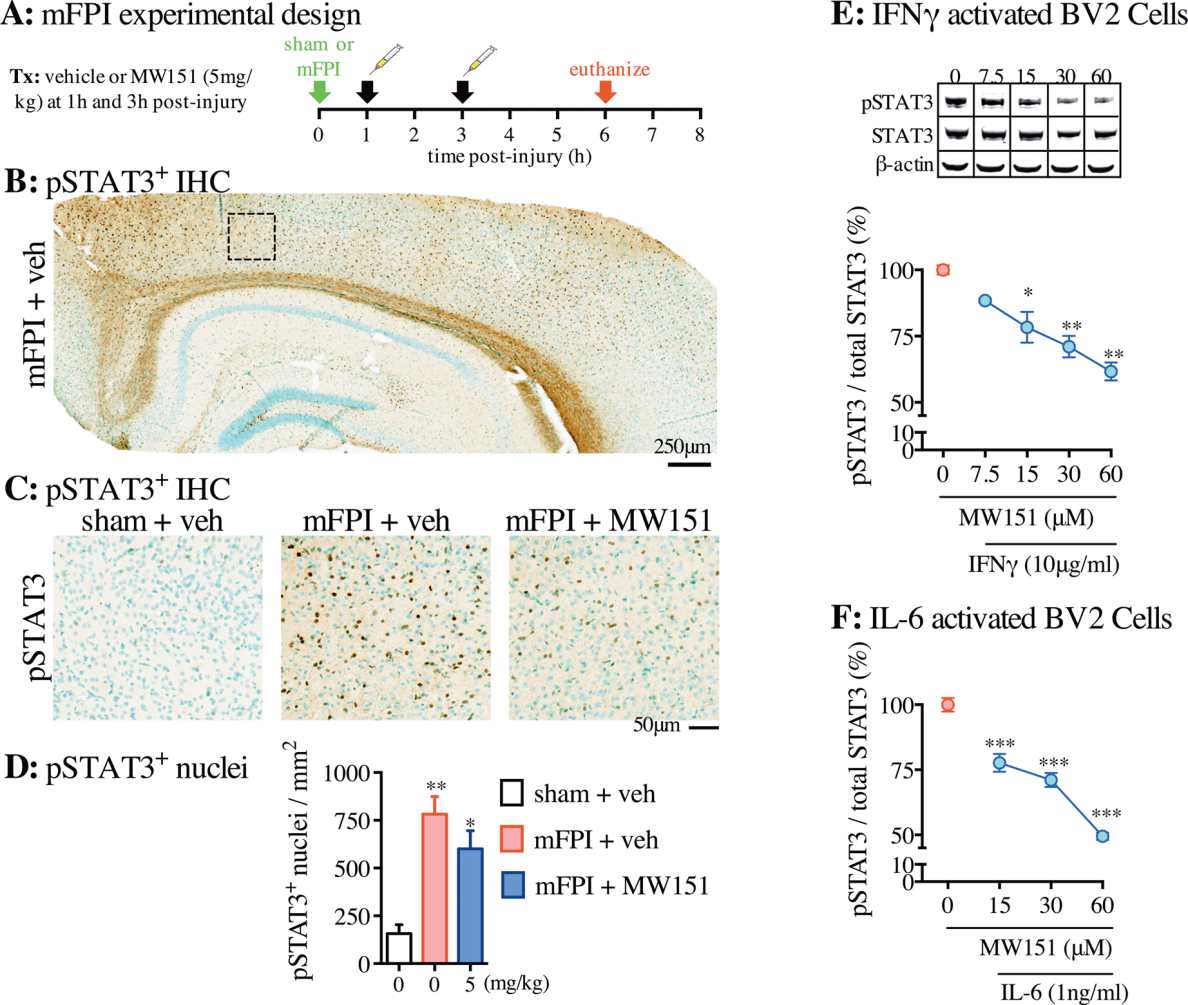
Effects of MW151 on pSTAT3. **(A)** Overview of *in vivo* TBI experimental design. (**B**) Representative example of pSTAT3 immunohistochemistry (IHC) in mFPI + veh -treated mice. Box indicates region shown at higher magnification in (**C**). Brown DAB staining is pSTAT3. Blue-green staining is a Methyl green counter stain. **(D)** Digital neuropathological quantification of pSTAT3^+^ nuclei in the cortex was done using the Aperio ScanScope and nuclear algorithm (n = 4 sham + veh, n = 9 mFPI + veh, n = 10 mFPI + MW151) (F_2,22_ = 7.5286; p = 0.0037). *p<0.05, **p<0.001 compared to mFPI + veh. (mFPI = midline fluid percussion injury; veh = vehicle). **(E)** BV-2 cells were treated with veh or MW151 and stimulated with IFNγ (10μg/ml) for 60min, then cell lysates were harvested for western blot analysis. The data presented is a representative experiment (n = 2–3 samples per group), with the experiment replicated 3 times. (*p<0.05, **p<0.01 compared to IFNγ + veh). **(F)** BV-2 cells were treated with veh or MW151 and stimulated with IL-6 (1ng/ml) for 60min, then cell lysates were harvested for ELISA. The data presented is a representative experiment (n = 4–6 samples per group), with the experiment replicated 4 times. (***p<0.0001 compared to IL-6 + veh).

A previous report [36], studying a different type of brain injury and using an analog of MW151, suggested that signal transducer and activator of transcription 3 (STAT3) might be a potential molecular reporter of pharmacological action for this potential first-in-class type of therapeutic glial modulator. To explore this possibility in the diffuse injury model with MW151 treatment, we examined brain tissue from saline and MW151 -treated mFPI mice for pSTAT3 staining, an end point surrogate for STAT3 activity. This is a repeat administration of MW151 at 5mg/kg (experimental outline in **Fig 6C**). Staining for pSTAT3 in mFPI injured mice is evident throughout the cortex and into the hippocampus (**Fig 6D)**. Comparisons of sham injured mice to the treatment control group (mFPI + veh) and MW151 treatment group (mFPI + MW151) reveal an increase in pSTAT3 positive cells after mFPI that is attenuated in mice treated with MW151 (**Fig 6E**). Digital neuropathological quantification of pSTAT3 positive nuclei in the cortex demonstrated an increase in pSTAT3 positive nuclei in the mFPI mice (mFPI + veh, p = 0.0009; mFPI + MW151, p = 0.0111) compared to sham + veh -treated mice (**Fig 6F**). The mFPI + MW151 -treated mice showed a 23% reduction in pSTAT3 positive nuclei (p = 0.1575) compared to the mFPI + veh -treated mice. However, repeat administration of a maximal dose of MW151 did not reduce pSTAT3 back to sham levels (**Fig 6F**).

In order to explore further the potential of pSTAT3 as a quantitative reporter for glia engagement by MW151, we evaluated the concentration-dependent effect of MW151 on pSTAT3 status in BV-2 cells stimulated with IFNγ or IL-6, which are inflammatory cytokines associated with adverse outcomes following TBI, and known activators of STAT3 pathway. BV-2 cells were stimulated with IFNγ or IL-6 for 60 min in the absence or presence of increasing concentrations of MW151. Western blot analysis of pSTAT3, STAT3, and β-actin were done on harvested cells. MW151 inhibited pSTAT3 levels in a concentration-dependent manner in BV-2 cells stimulated with either IFNγ (**Fig 6A**) or IL-6 (**Fig 6B**). The concentration-dependent effects of MW151 on BV-2 cells supports the above findings with brain tissue sections from mFPI mice. While the results do not provide evidence for a specific molecular target for MW151, they do support the hypothesis that pSTAT3 might be a reporter for MW151 pharmacological engagement.

## Discussion

The results presented here extend the potential clinical utility of MW151 to diffuse brain injury and support a selective glial action as one component of MW151 pharmacological action in acute brain injury. Microglia exhibit an array of responses in their physiological role in homeostasis and in their pathophysiological role in disease progression. The results reported here for diffuse axonal injury add to a broad body of knowledge from diverse injury paradigms that show a consistent trend of MW151 attenuation of injurious proinflammatory cytokine production as one mechanism of pharmacological action. In addition, the results show that MW151 can bring about the disease modification with retention of normal glial processes when effective dosing is employed. Dosing is the pharmacological basis of therapeutic action, and a fundamental tenet of pharmacological dosing is that all drugs will eventually demonstrate adverse effects at some concentration or time window. In this regard, the selective *in vivo* and *in vitro* pharmacological actions of MW151 provide risk reduction related to selective modulation of innate immunity mechanisms, and add to an accumulating body of evidence from multiple disease and injury models for its viability as a potential disease modification mechanism.

MW151 is one deliverable from a function-based drug discovery approach that targeted pathophysiology progression mechanisms involving stressor-induced upregulation of neuroinflammatory responses [13]. The comparatively unbiased approach used an Alzheimer’s disease (AD)-relevant *in vivo* screen, and attenuation of injury-induced upregulation of proinflammatory cytokine production that was associated with synaptic dysfunction. The rationale was that inflammatory cytokines, IL-1β being an archetype, are important in maintaining optimal neuronal functions and homeostasis, but can contribute to pathophysiology progression or susceptibility to injury, with overproduction. In an amyloid beta (Aβ) infusion model where early proinflammatory cytokine level dysfunction was validated as a contributor to Aβ induced synaptic dysfunction, MW151 administered once daily at 2.5mg/kg for 2 weeks resulted in a 73% suppression of IL-1β. This pharmacodynamics effect on IL-1β levels correlated with nearly complete recovery of neuronal and synaptic dysfunction markers and cognitive function [13]. No adverse events were observed in a repeat administration or an acute dose escalation administration up to 20–50 times efficacy doses. Overall, the discovery approach was unbiased in terms of molecular targets but was heavily biased in terms of safety and pharmacological action focused on an established pathophysiology progression mechanism involving proinflammatory cytokines. The findings presented here for glial endpoint selectivity in an acute axonal injury model and *in vitro* glial cell culture studies are consistent with the outcomes from the discovery approach and the pharmacological profile of MW151.

The pharmacodynamics and efficacy of MW151 seen in the discovery approach were reinforced and extended in more detailed studies using genetically modified mouse AD models, and are also consistent with the effects on IL-1β levels reported here. For example, repeat MW151 administration to an APP/PS1 knock-in (KI) mouse model for 5 months (3 times per week starting at 6 months of age) resulted in 31% suppression of IL-1β levels compared to control vehicle-treated mice [19]. The levels of IL-1β in the mice treated with MW151 for 5 months were at approximately 3% of WT mice in the same study [19], but never below basal levels. Therefore, MW151 can attenuate injurious increases in IL-1β levels in AD-relevant models close to basal levels, but does not suppress below basal levels. Similar homeostatic properties are seen in non-AD models. Most relevant to the current study, post-injury administration of MW151 (5mg/kg) in two closed head TBI mouse models reduced IL-1β levels by approximately 50% compared with concomitant improvement in neurologic outcome, but did not suppress IL-1β below basal levels [4, 20]. Taken in its entirety across diverse animal models of brain injury and disease progression, MW151 can attenuate injurious increases in IL-1β production toward a homeostatic level with positive neurologic outcomes without adverse events or immunosuppression of basal IL-1β production. The selectivity of MW151 on glial physiological responses reported here is consistent with this trend.

Clearly, dosing will determine if an injury or disease model responds well to low doses of MW151, and how various glial changes are related to efficacy and safety. For example, MW151 suppressed IL-1β levels back to basal in the AD-relevant APP/PS1 KI mice whether administration was for 1 week or 5 month duration, but IBA1 staining was only suppressed with 5 month of treatment [19]. In contrast, our results here in the mFPI model revealed a slight increase in IBA1^+^ staining in the MW151-treated mice, but our previous studies of a CHI model of TBI showed that MW151 treatment suppressed IBA1^+^ staining [4, 19]. The theme of IBA1 staining association with the *in vivo* pharmacodynamics endpoint of cytokine levels or with pharmacological safety and neurologic outcomes is not clear at this time. Regardless, we did not observe a major effect on the *in vitro* cellular functions of glia proliferation, migration, or phagocytosis.

The clear MW151 pharmacodynamic endpoint of IL-1β levels in the cortex across multiple models and injury mechanisms is well linked to efficacy as measured by synaptic markers and hippocampal-mediated cognitive function. This is consistent with the fact that brain IL-1β level was one of the pharmacodynamic endpoints used for the unbiased functional discovery approach. There is a need to move back into the cellular and molecular mechanisms involved in the efficacy-linked IL-1β pharmacological response. However, the use of a functional approach requires anticipation of potential multiple targets or pathways involved in pharmacodynamics endpoint changes or in neurologic efficacy. Regardless, a report [36] using an analog of MW151 noted a change in STAT3 in a CHI model, warranting the investigation reported here. As reported here, analysis of brain tissue from mFPI animals revealed an increase in pSTAT3 staining, and decreased staining in the cortex of MW151-treated animals. The indication of an *in vivo* change was confirmed by an *in vitro* cell line experiment using BV-2 cells and their stimulation by known activators of the STAT3 pathway, interferon (IFNα and IFNγ) and IL-6 mediated signaling [37–39]. MW151 brings IL-1β levels and cognitive function to basal or control levels (here and previous reports), but does not bring pSTAT3 levels below 50% in either *in vivo* or *in vitro* assays. The results are not fully supportive of STAT3 or upstream modifiers of STAT3 as molecular targets for MW151, but support an axis involving STAT3 as being involved in the pharmacological action of MW151. However, the concentration-dependent effect indicates that pSTAT3 might be worthy of pursuit as a pharmacodynamic marker related to the pharmacological mechanism that is also relevant to control of innate immunity.

Overall, our results expand the potential utility of MW151 to a mild-to-moderate diffuse brain injury model, and illustrate that overproduction of IL-1β can be therapeutically targeted while sparing other reactive glial responses to injury.

## Abbreviations

AD: Alzheimer’s disease
Aβ: amyloid beta
CHI: closed head injury
CNS: central nervous system
CytD: cytochalasin D
IHC: immunohistochemistry
IL-1: interleukin-1
MSD: Meso Scale Discovery
mFPI: midline fluid percussion injury
STAT3: signal transducer and activator of transcription 3
TBI: traumatic brain injury
veh: vehicle
